# Spatial-Temporal Evolution of Health Impact and Economic Loss upon Exposure to PM_2.5_ in China

**DOI:** 10.3390/ijerph19041922

**Published:** 2022-02-09

**Authors:** Xialing Sun, Rui Zhang, Geyi Wang

**Affiliations:** School of Management, China University of Mining and Technology (Beijing), Beijing 100083, China; bqt1900501005@student.cumtb.edu.cn (X.S.); sqt1900501012@student.cumtb.edu.cn (G.W.)

**Keywords:** PM_2.5_, health impact, economic loss, spatial-temporal evolution

## Abstract

Exposure to PM_2.5_ can seriously endanger public health. Policies for controlling PM_2.5_ need to consider health hazards under different circumstances. Unlike most studies on the concentration, distribution, and influencing factors of PM_2.5_, the present study focuses on the impact of PM_2.5_ on human health. We analysed the spatial-temporal evolution of health impact and economic loss caused by PM_2.5_ exposure using the log-linear exposure-response function and benefit transfer method. The results indicate that the number of people affected by PM_2.5_ pollution fluctuated and began to decline after reaching a peak in 2014, benefiting from the Air Pollution Prevention and Control Action Plan. Regarding the total economic loss, the temporal pattern continued to rise until 2014 and then declined, with an annual mean of 86,886.94 million USD, accounting for 1.71% of China’s GDP. For the spatial pattern, the health impact and economic loss show a strong spatial correlation and remarkable polarisation phenomena, with high values in East China, North China, Central China, and South China, but low values in Southwest China, Northwest China, and Northeast China. The spatial-temporal characterisation of PM_2.5_ health hazards is visualised and analysed accordingly, which can provide a reference for more comprehensive and effective policy decisions.

## 1. Introduction

Air pollution is the fourth leading cause of death globally, preceded by poor diet, high blood pressure, and smoking risks [1]. PM_2.5_ is the primary pollutant in the atmosphere and is defined as particulate matter with an aerodynamic equivalent diameter of less than or equal to 2.5 microns in ambient air, consisting mainly of organic carbon (OC), elemental carbon (EC), nitrate, sulphate, ammonium and sodium salts, etc. Compared with coarser atmospheric particulate matter, PM_2.5_ has a small particle size, large area, strong activity, is easily accompanied by toxic and harmful substances, and has a long residence time and long transport distance in the atmosphere, thus having a greater impact on atmospheric environmental quality and human health. Furthermore, PM_2.5_ can directly enter the bronchi and alveoli through breathing, quickly enter the blood circulation and spread throughout the whole body, leading to an increase in morbidity and mortality [2,3,4]. Epidemiological studies have shown that PM_2.5_ exposure ranks the highest among the environmental risk factors affecting the health of residents [5]. Therefore, effective PM_2.5_ control strategies are the key to achieving sustainable development goals and urban public health all over the world [6,7,8].

As China is the largest developing country in the world, the PM_2.5_ produced by urbanisation and industrialisation is a constraint of social welfare [9,10,11]. To alleviate such severe PM_2.5_ pollution, the State Council issued the Air Pollution Prevention and Control Action Plan in 2013, proposing ten strict air pollution prevention and control actions. China has remarkably improved urban air pollution control. The average annual concentration of PM_2.5_ in China decreased from 58 µg/m^3^ in 2013 to 33 µg/m^3^ in 2020 [5], and the number of deaths caused by PM_2.5_ dropped significantly during 2013–2017 [12]. However, the PM_2.5_ pollution in China is still higher than that in developed countries, and severely polluted air with PM_2.5_ as the primary pollutant frequently occurs in many regions. There are 48 Chinese cities among the 100 cities globally with the most severe air pollution. In addition, among the 400 cities in mainland China, only 53% of the cities have an annual average PM_2.5_ concentration that meets China’s second level of air quality standard (35 µg/m^3^), and only 2% of the cities have lower PM_2.5_ than the standard value (10 µg/m^3^) set by the World Health Organization (WHO) [13,14].

Studies on PM_2.5_ can be grouped into two categories. The first comprises the hottest and most numerous, and they mainly start from a macro perspective, focusing on physical cause analysis, meteorological characteristics, concentration measurement, influence factors, control pathways, etc. [15,16,17,18,19]. The second category comprises micro pathological analysis from a medical perspective and exposure risk analysis from a health statistics perspective [20,21,22,23]. The present study focuses on the PM_2.5_ health hazards to urban residents from an exposure risk perspective. Regarding health hazard assessment, Bu et al. [24] demonstrated that 4.58 million deaths and 142.52 million disability-adjusted life years were caused by PM_2.5_ exposure globally in 2017, and the increasing pollution will increase the burden on the health of older people and low-income groups. Long-term exposure to environments polluted by PM_2.5_ has increased the global disease burden and is an obstacle to economic prosperity [25]. For China’s PM_2.5_ impact assessment, Maji et al. [26] pointed out that the health cost of diseases caused by PM_2.5_ and PM_10_ pollution in 190 cities in China was approximately 304,122 million USD from 2014–2015, accounting for 2.94% of GDP. Xie et al. [27] demonstrated that the GDP loss caused by PM_2.5_ pollution in China will reach 2%, and the health expenditure will reach 25.2 billion USD by 2030 without implementing appropriate control strategies for PM_2.5_. Yue et al. [12] found that if China adopts more stringent air quality targets and reaches a PM_2.5_ concentration of 10 µg/m^3^, the number of deaths from PM_2.5_ will drop by 43.3%.

In addition to the above national-level studies, most studies on the health impact and economic loss of PM_2.5_ pollution in China have focused on specific cities, regions, or industry sectors [21,28,29,30,31]. For example, Li et al. [32] predicted the PM_2.5_ emission caused by energy consumption in Beijing in the future, and calculated the resultant health impact and economic loss. Chen et al. [29] focused on the public health effect and its economic loss upon exposure to PM_2.5_ generated by coal consumption in 2015; precisely, the impact of PM_2.5_ was estimated from the perspective of the industry, and an attempt was made to put forward a total coal consumption control scheme. Wang et al. [33] improved the traditional PM_2.5_-related health loss assessment model and estimated the health-related economic loss of Beijing-Tianjin-Shijiazhuang in 2017 and 2018 based on this improved model.

The majority of the extant studies cannot fully reflect the overall situation and regional differences in human health and socioeconomic impact of PM_2.5_ pollution in China, and lack temporal and spatial distribution analysis. Some studies have tried to make up for the deficiency of the analyses of the temporal and spatial distribution, but due to the PM_2.5_ data not being available from ground monitoring stations until December 2013, there is a lack of long-term dynamic evolution analysis. For example, Guan et al. [34] emphasised the distribution and temporal differences of health loss in 338 cities in China, but the time range of the study was only limited to 2015–2017. Similarly, Maji et al. [35] only analysed the spatial distribution differences of health loss in 338 cities in China in 2016. It is worth noting that apart from the annual differences, due to the differences in PM_2.5_ concentration and human activities in each season or even month, the health impact changes significantly in different seasons and months. Yang et al. [36] emphasised the significant difference in PM_2.5_-related health impact and economic loss in summer and winter, pointing out that the existing research about the seasonal and monthly changes on the health impact of PM_2.5_ is insufficient.

Given the background, this study aims to assess the health impact and economic loss caused by PM_2.5_ pollution and depict their spatial-temporal evolution in 30 provinces of China from 2005–2017. The first contribution of this study is to identify the time-dynamic evolution in a long sequence based on satellite remote sensing data, which is superior to the ground detection statistics started in December 2013, and there are no problems of insufficient monitoring points and artificial fraud. The second contribution is that a more accurate assessment using the log-linear exposure-response function and benefit transfer method, the dynamic evolution analysis of the kernel density, and uncertainty analysis for different baselines are analysed. The third contribution of this study is to analyse and visualise regional differences and the seasonal and monthly differences for different endpoints on the health impact and economic loss of PM_2.5_ exposure. The combination of spatial and temporal analysis offers a profound revelation of the distribution difference and evolution of health impact and economic loss at the provincial level during different development periods of China. Altogether, we focus on the differences between the country as a whole and on the 30 provinces to propose more effective environmental protection in terms of regional coordination and to provide empirical data to support the prevention and control of PM_2.5_ at the national level. The results are helpful for the formulation of PM_2.5_ management and public health policies in line with China’s national conditions and geospatial characteristics.

The remainder of this paper is organised as follows. The second part comprises the methods and data sources, wherein the methods of health impact assessment and health economic loss calculation are introduced, and the corresponding data sources are explained. The third part describes the results, which show the spatial-temporal evolution and seasonal differences of the health impact and economic loss, and the dynamic evolution of kernel density. The fourth part comprises the conclusion and policy implications.

**Figure 1:**
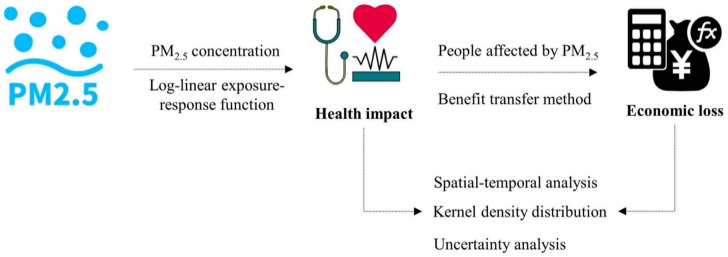
The research framework.

The research framework can be summarised as in Figure 1.

## 2. Methods and Data Sources

### 2.1. Health Impact Assessment

In this study, we used the relative risk based on epidemiology to estimate the long-term health impact caused by exposure to PM_2.5_; specifically, the number of deaths or illnesses. According to the disease classification of ICD-10 [37], and as per Yin et al. [38], we divided the health endpoints into all-cause mortality, respiratory mortality, cardiovascular mortality, lung cancer mortality, respiratory hospital admission, cardiovascular hospital admission, chronic bronchitis, acute bronchitis, and asthma attacks; these were classified as mortality, hospital admission, and outpatient service. The harm of PM_2.5_ to the human body is omnidirectional, especially in respiratory, cardiovascular, and lung disease. Referring to Chen et al. [39] and Guo et al. [40], for every 10 µg/m^3^ increase in the mean concentration of PM_2.5_, the all-cause mortality, respiratory mortality, cardiovascular mortality, and lung cancer mortality increased by 0.25%, 0.48%, 0.25%, and 2.48%, respectively. Therefore, this study focuses on respiratory mortality, cardiovascular mortality, and lung cancer mortality among the all-cause mortality caused by PM_2.5_.

The exposure-response function reveals the relationship between changes in the PM_2.5_ concentration and health impact. In previous studies, three exposure-response functions were used: integrated exposure risk function (IER), log-linear function (LL), and nonlinear power law function (NLP) [35]. The LL is more suitable for the health impact assessment in high-particulate-matter pollution areas [41], and has been widely used in previous studies [21,26,33,38]. The relative risk based on the log-linear exposure-response function can be expressed as Equation (1):

(1) $$RR_{s,i}=\exp[\beta_{s}(C_{i}-C_{0})]$$

where s is the health endpoint, i is the province, $RR_{s,i}$ is the relative risk of PM_2.5_ exposure, $\beta_{s}$ is the exposure-response coefficient based on Chinese epidemiology, that is, the impact of 1 µg/m^3^ PM_2.5_ concentration increment on the incidence of the s health endpoint; $C_{i}$ is the annual average daily PM_2.5_ concentration in the i province; $C_{0}$ is the threshold of PM_2.5_ concentration, wherein the PM_2.5_ concentration below this threshold is assumed to have no impact on health.

The health impact of PM_2.5_ exposure can be calculated by Equation (2) [35]:

(2) $$HI_{s,i}=[(RR_{s,i}-1)/RR_{s,i}]\times B_{s}\times EP_{i}$$

where $HI_{s,i}$ is the number of deaths or illnesses, and it is used to characterise the health impact. $B_{s}$ is the baseline incidence of the s health endpoint, and $EP_{i}$ is the number of people exposed to PM_2.5_ in the i province, expressed by the permanent population at the end of the year.

The exposure-response coefficient β (µg/m^3^) and baseline incidence B of each health endpoint are presented in Table 1 and Table 2, respectively.

### 2.2. Economic Loss Estimation

The health impact of PM_2.5_ exposure was monetised to assess its economic loss. As the yearly health costs of different health endpoints in different provinces are unavailable, the benefit transfer method (BTM) is used to evaluate the economic loss of each health endpoint in 30 provinces by year (Equation (3)) [35,48,49].

(3) $$HC_{s,i(t)}=HC_{s,k}\times {\left(\frac{G_{i,k}}{G_{k}}\right)}^{\alpha }\times {\left(1+\%\Delta P_{i}+\%\Delta G_{i}\right)}^{\alpha }$$

where s is the health endpoint (s=1,⋯,9), i is the province (i=1,⋯,30), t is the year, k is the base year (2005), $HC_{s,i(t)}$ is the adjusted unit economic loss, and $HC_{s,k}$ is the per unit economic loss of the s health endpoint in China in the base year. The unit economic losses of each health endpoint in the base year are presented in Table 3. $G_{i,k}$ is the per capita GDP calculated by the purchasing power parity (PPP); $\%\Delta P_{i}$ is the percentage increase or decrease in the consumer price index of the i province from k year to t year; $\%\Delta G_{i}$ is the percentage increase or decrease in the per capita GDP of the i province from k year to t year; and α is the income elasticity of the economic loss, which is 0.8, as recommended by the OECD [50].

The total economic loss $EC_{s,i(t)}$ of the s health endpoint in i province in the t year can be calculated by Equation (4).

(4) $$EC_{s,i(t)}=HC_{s,i(t)}\times HI_{s,i(t)}$$

where $HC_{s,i(t)}$ is the adjusted unit economic loss of the s health endpoint in the i province; and $HI_{s,i(t)}$ is the number of deaths or illnesses of the s health endpoint in the i province calculated by Equation (2).

### 2.3. Data Sources

We used the data of 30 province-level regions in China from 2005 to 2017 as the research sample. Due to some indicator data not being available, we did not study Hong Kong, Macao, Taiwan, and Tibet. The 30 provinces and their eastern, central, and western divisions are as follows: the eastern regions include Beijing, Tianjin, Hebei, Liaoning, Shanghai, Jiangsu, Zhejiang, Fujian, Shandong, Guangdong, and Hainan; the central regions include Shanxi, Jilin, Heilongjiang, Anhui, Jiangxi, Henan, Hubei, and Hunan; and the western regions include Inner Mongolia, Guangxi, Chongqing, Sichuan, Guizhou, Yunnan, Shaanxi, Gansu, Qinghai, Ningxia, and Xinjiang. The PM_2.5_ data were obtained from the NASA Earth Observation raster data from the Socioeconomic Data and Applications Centre (SEDAC) of Columbia University in the United States. ArcGIS was used to parse the remote sensing data into comparable data in regional units. We adopted the standard value of 10 µg/m^3^ as the baseline concentration for PM_2.5_ [53]. The permanent population at the end of the year, GDP, and consumer price index were obtained from the China Statistical Yearbook 2006–2018 [54].

## 3. Results

### 3.1. Evaluating Health Impact

**Figure 2:**
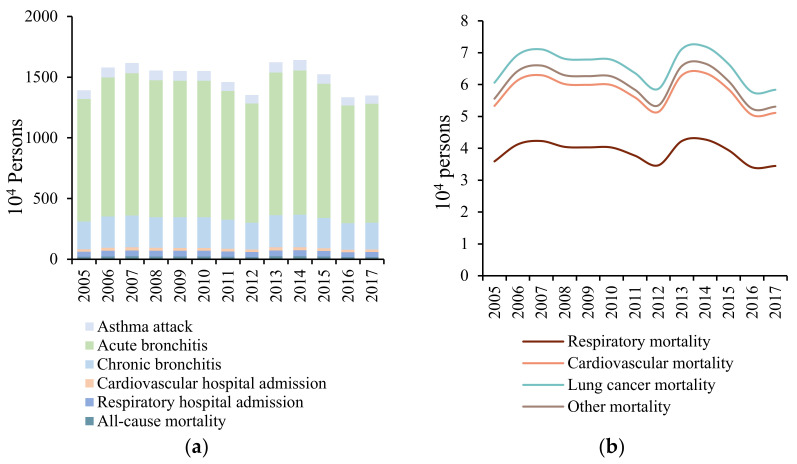
Annual changes in the health impact due to PM_2.5_ exposure. (**a**) Health impact assessment of PM_2.5_ exposure in China from 2005–2017. (**b**) Disease-specific mortality assessment due to PM_2.5_ exposure in China from 2005–2017.

Based on the log-linear exposure-response function, the health impact of PM_2.5_ exposure was calculated, and the results are shown in Figure 2a. For the temporal pattern, the total health impact of PM_2.5_ exposure fluctuates continuously, rising from 13.92 million in 2005 to a peak of 16.17 million in 2007. Subsequently, it shows a steady downward trend, reaching a minimum of 13.54 million in 2012, and increases sharply in 2013. In 2014, a maximum of 16.41 million was estimated, which gradually decreased to 13.50 million in 2017, with an annual average of 15.03 million. The sharp increase in the value of health impact in 2013 occurred due to the increased PM_2.5_ concentration caused by the high-intensity haze pollution in China. The total health impact continued to decline from 2014–2017. Thus, the State Council issued the Air Pollution Prevention and Control Action Plan in 2013, which has achieved positive results in haze control. From the perspective of different health endpoints, the number of outpatients far exceeds that of deaths and hospitalisations, among which the number of patients with acute bronchitis is the largest, accounting for 73% of the total health impact, followed by chronic bronchitis, accounting for 16%; the prevalence of asthma attacks is lower than that of the former two. The number of hospitalisations for respiratory and cardiovascular diseases does not account for a high proportion of the total health impact (less than 5% combined), but both are higher than the all-cause mortality.

Although the all-cause mortality of PM_2.5_ exposure accounted for the lowest proportion of the total health impact (approximately 1.5%) and the annual average death toll was 0.2227 million, the impact of mortality was much higher than that of the hospital admission and outpatient service. Therefore, the disease-specific mortality was further analysed (Figure 2b). Specifically, lung cancer mortality accounted for the majority of the all-cause mortality (more than 29%), followed by the cardiovascular and respiratory mortalities, accounting for nearly 26% and 17%, respectively. Yin et al. [38] calculated that lung cancer mortality, cardiovascular mortality, and respiratory mortality accounted for 32%, 25%, and 15% of the all-cause mortality, respectively, which is roughly consistent with the above results.

**Figure 3:**
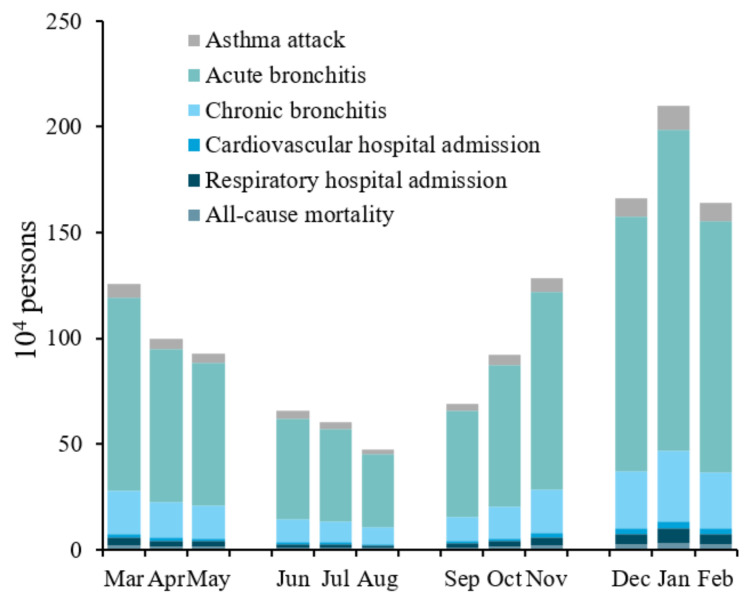
Health impact assessment due to PM_2.5_ exposure by month in 2017.

The health impact of PM_2.5_ exposure not only shows significant annual differences but also has distinct seasonal and monthly variations within the year. Figure 3 shows the health impact for each health endpoint by month due to PM_2.5_ exposure in 2017. The seasons were divided as follows: spring from March to May, summer from June to August, autumn from September to November, and winter from December to February. Overall, the health impact of PM_2.5_ exposure showed a U-shaped curve from spring to winter, and gradually decreased in winter, spring, autumn, and summer (in descending order of magnitude). In specific cases, the health impact in winter is 5.4062 million, about 3.12 times that in summer, and 3.19 million in spring, slightly higher than that in autumn. The health impact is the highest in winter, which may be due to the high PM_2.5_ concentration caused by the increase in heating coal and meteorological conditions that are unfavourable to the diffusion of air pollutants [55]. Yang et al. [36] estimated that the amount of mortality or morbidity caused by PM_2.5_ pollution in 28 Chinese cities during the winters of 2013–2016 was about three times that in the summer, which is consistent with the above results. The monthly cases of health impact were the highest in January, reaching 2.10 million, and the lowest in August at 0.47 million.

**Figure 4:**
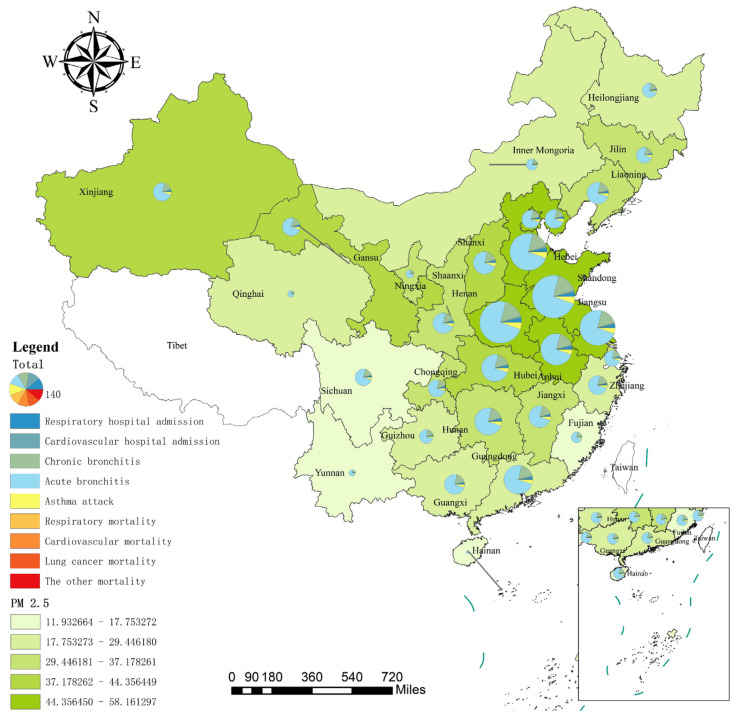
Spatial distribution of the health impact due to PM_2.5_ exposure by province in 2017. (Notes: a. The 9 health endpoints and their respective colours are explained in the Legend. The pie chart represents the total health impact, and the green graph at the bottom is the PM_2.5_ value of each province. b. Owing to the space limitations, the spatial distribution map of other years is not shown; this can be obtained from the corresponding author.).

Figure 4 shows the spatial distribution of the health impact due to PM_2.5_ exposure at different health endpoints by province in 2017. The PM_2.5_ quintile map at the bottom of Figure 4 shows that the PM_2.5_ concentration of each province has obvious spatial distribution characteristics. The PM_2.5_ concentration in East and North China was the highest, followed by Central and South China, and the lowest was observed in Southwest China, Northwest China, and Northeast China. The distribution of PM_2.5_ concentration is consistent with the conclusion of Li and Ye [56]. Firstly, East China and North China are densely populated, and the traffic and industrial pollution along with the rapid economic development led to an increase in PM_2.5_ concentration in these regions. For example, the annual average PM_2.5_ concentration is above 45 µg/m^3^ in the Tianjin, Henan, Hebei, Shandong, Anhui, Jiangsu, Beijing, and Shanxi provinces. Secondly, there are relatively few heavy-polluting industries in Central and South China, and their PM_2.5_ concentration is lower than that in East and North China. For example, the Hubei province in Central China has an annual average daily PM_2.5_ concentration of 40.64 µg/m^3^. Moreover, South China is located on the west coast of the Pacific Ocean. The clean and humid sea breeze brought by the southeast winds in summer and the warm current from Japan cause the atmospheric pollutants emitted by economic and development activities in this region to be diluted; thus, the PM_2.5_ concentration in this region is not high [57,58]. Thirdly, the economic foundation of Southwest China and Northwest China is weak, and the pollution emission due to the social and economic activities of these regions with low population density is not far beyond the self-purification and repair capacity of the atmospheric environment. Moreover, the southwest and northwest regions are mostly deserts and plateaus, and the northwest winds from Siberia in winter promote air mobility in these regions, resulting in a low PM_2.5_ concentration. For example, the PM_2.5_ concentration in Inner Mongolia, Yunnan, and Guizhou are low, but the PM_2.5_ concentration in Gansu and Xinjiang (about 43 µg/m^3^) in the northwest is higher than that in the surrounding areas. The concentration of PM_2.5_ in Northeast China is also relatively low. For example, the annual average concentration of PM_2.5_ in Heilongjiang is 22.68 µg/m^3^.

The overall spatial distribution pattern of the health impact upon exposure to PM_2.5_ is as follows: that in East China, North China, Central China, and South China is higher, while that in Southwest China, Northwest China, and Northeast China is lower. Provinces with high PM_2.5_ concentrations in East China, North China, and Central China have a relatively high health impact caused by PM_2.5_. For example, Shandong had an average annual PM_2.5_ concentration of 57.89 µg/m^3^ in 2017, and the total health impact reached 1.66 million, ranking first in China, followed by Henan at 1.60 million. Although the annual average concentration of PM_2.5_ in South China is not high, Guangdong in South China is densely populated, with a mortality and morbidity of 0.76 million, ranking sixth in China. The total health impact is relatively low in Southwest, Northwest, and Northeast China, such as Ningxia, Jilin, Qinghai, Yunnan, Heilongjiang, Guizhou, and Inner Mongolia, which benefit from the low PM_2.5_ concentration and the low total population in these regions. From the perspective of different health endpoints, the all-cause mortality in Hebei, Jiangsu, Anhui, Shandong, Henan, and Guangdong exceeded 10,000, which is higher than the total health impact in Hainan (8400). Thus, exposure to high PM_2.5_ concentrations has caused severe mortality losses in such traditionally polluting industry-intensive provinces.

### 3.2. Estimating Economic Loss

**Figure 5:**
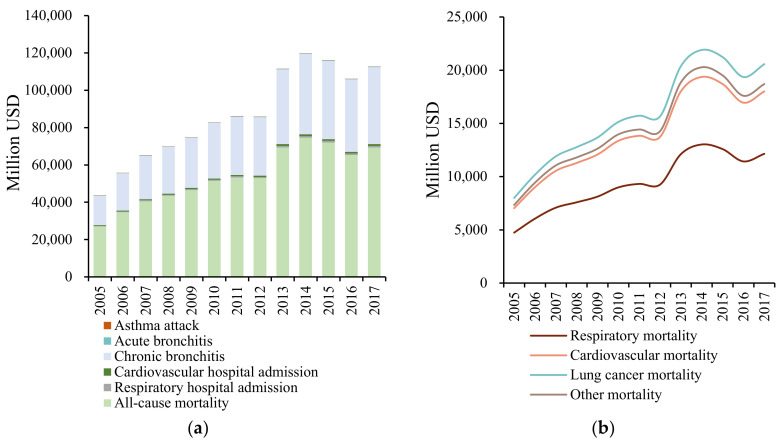
Annual changes in the economic loss by PM_2.5_ exposure. (**a**) Estimation of economic loss by PM_2.5_ exposure in China during 2005–2017. (**b**) Estimation of the disease-specific mortality economic loss by PM_2.5_ exposure in China during 2005–2017.

For the temporal pattern, the economic loss caused by PM_2.5_ exposure shows an upward trend, with fluctuations from 2005–2017 (Figure 5a). The total economic loss has increased from 43,775.81 million USD in 2005 to 86,041.52 million USD in 2011 with a declining growth rate. In 2012, a slight decrease occurred, but it surged at a growth rate of 29.89% in 2013, going past 100 billion USD. It rose to a peak of 119,684.29 million USD in 2014, decreased slightly in 2015 and 2016, and increased subsequently in 2017 to 112,668.02 million USD. The average annual value of the total economic loss is 86,886.94 million USD, accounting for 1.71% of the GDP of China.

The economic loss of each health endpoint differs from the health impact. Specifically, the economic loss of the all-cause mortality accounts for approximately 62% of the total economic loss, but the proportion of all-cause mortality in the total health impact is below 1.5%. The economic loss of chronic bronchitis accounts for approximately 36% of all cases, followed by all-cause mortality. Although acute bronchitis accounts for the highest proportion of the total health impact (about 72%), its economic loss accounts for only 0.2% of the total. Therefore, on the one hand, preventing the impact of acute bronchitis with high rates of incidence on large crowds is necessary; on the other hand, reducing the economic loss caused by mortality and chronic bronchitis due to PM_2.5_ is also required.

The economic loss of disease-specific mortality due to PM_2.5_ exposure is shown in Figure 5b. The total economic loss of all-cause mortality has increased since 2005, reaching a maximum of 74,560.80 million USD in 2014. It declined after 2014 and reached 69,409.91 million USD in 2017. The average annual economic loss caused by all-cause mortality is 53,899.77 million USD. Among them, the economic loss due to lung cancer mortality is the highest at 15,883.78 million USD, followed by cardiovascular mortality at 13,993.43 million USD, and respiratory mortality at 9417.80 million USD. The sum of these accounts for about 73% of the total economic loss. During the study period, the economic loss caused by mortality showed a fluctuating upward trend, implying that PM_2.5_ pollution not only affects the safety and health of residents, but also results in social and economic losses due to premature death.

**Figure 6:**
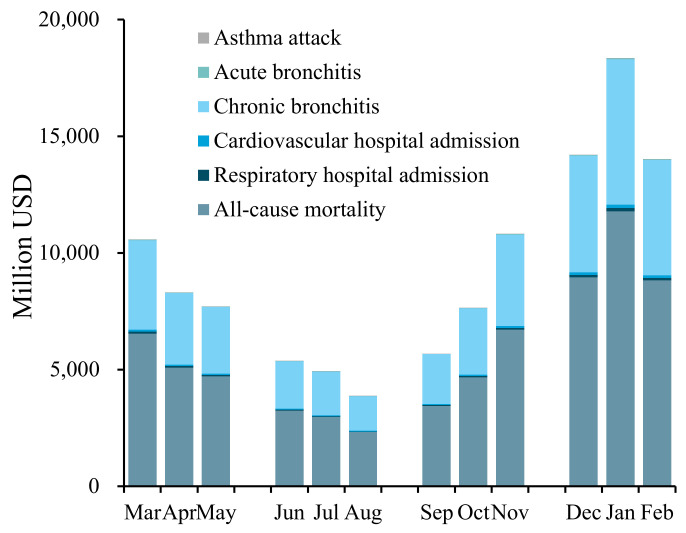
Estimation of the economic loss due to PM_2.5_ exposure by month in 2017.

The changes in economic losses during the year are shown in Figure 6. On the whole, the health-related economic loss in each month from spring to winter shows a U-shaped curve. The trend of this curve is consistent with that of the monthly health impact curve. The economic loss was the lowest in August (approximately 3863.18 million USD), and highest in January (about 18,333.49 million USD). Notably, the economic loss in January was 4.75 times that in August, and 3.42 and 3.73 times that in June and July, respectively. This monthly difference mainly occurs due to the difference in PM_2.5_ concentration, which further proves the significant impact that PM_2.5_ concentration has on economic loss. In terms of the health endpoints, all-cause mortality and chronic bronchitis are the most important sources of economic loss, and the sum of the economic losses caused by them is more than 98% every month.

**Figure 7:**
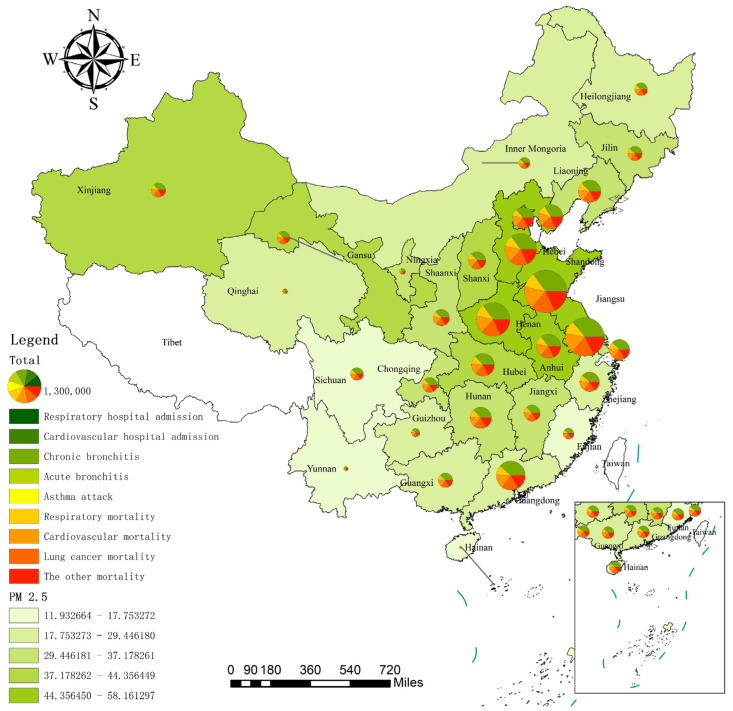
Spatial distribution of economic loss due to PM_2.5_ exposure by province in 2017. (Notes: a. The 9 health endpoints and their respective colours are explained in the Legend. The pie chart represents the total economic loss, and the green graph at the bottom is the PM_2.5_ value of each province. b. Owing to the space limitations, the spatial distribution map of other years is not shown; this can be obtained from the corresponding author.).

The spatial distribution of the economic losses of different health endpoints in each province is shown in Figure 7. Overall, the economic losses are high in East, North, Central, and South China, and low in Southwest, Northwest, and Northeast China. Specifically, the economic loss by PM_2.5_ exposure is relatively significant in Shandong, Jiangsu, and Anhui in East China, Hebei and Tianjin in North China, Henan, Hubei, and Hunan in Central China, and Guangdong in South China. However, the economic loss is relatively small in Sichuan, Guizhou, and Yunnan in Southwest China, Gansu, Qinghai, Ningxia, and Xinjiang in Northwest China, and Heilongjiang and Jilin in Northeast China.

The total economic loss in six heavily polluted provinces of Shandong, Jiangsu, Henan, Hebei, Guangdong, and Tianjin is 61,338.97 million USD, which is more than half of the national total economic loss of 112,668.00 million USD. This implies that the PM_2.5_ pollution caused by the economic development in these provinces has seriously endangered the health of residents and has generated substantial economic losses. In comparison, Guangdong ranks first in the GDP and population in the country, but its economic loss caused by PM_2.5_ pollution is significantly lower than that of Shandong, Jiangsu, Henan, and Hebei. Simultaneously, it can be seen that Shandong suffered the highest economic loss, amounting to 16,061.91 million USD, accounting for 2.14% of the regional GDP, while Hainan suffered the lowest economic loss, amounting to 52.23 million USD, accounting for 0.13% of the regional GDP. Interestingly, the economic losses of all-cause mortality and chronic bronchitis of Shandong are 10,231.29 million USD and 5542.05 million USD, respectively. Both of these exceed the sum of economic losses of Gansu, Inner Mongolia, Fujian, Guizhou, Ningxia, Qinghai, Yunnan, and Hainan, and are 100 times more than that of Hainan. This further confirms that lung cancer mortality, respiratory mortality, cardiovascular mortality, and chronic bronchitis due to PM_2.5_ are the main sources of health and economic loss in each province.

### 3.3. Dynamic Evolution Analysis of the Kernel Density

**Figure 8:**
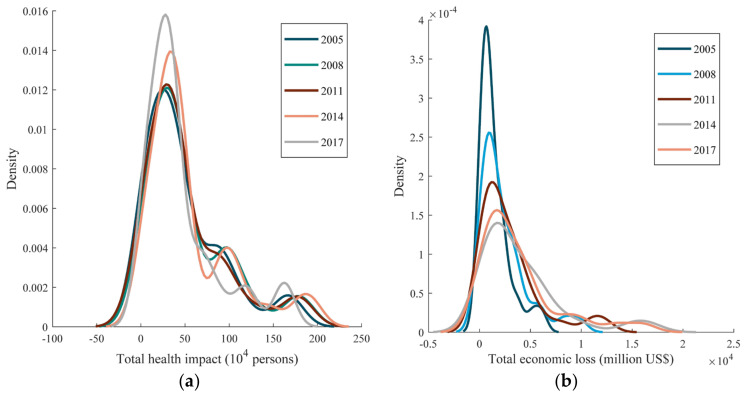
Kernel density analysis of the health impact and economic loss by PM_2.5_ exposure. (**a**) Kernel density analysis of the health impact. (**b**) Kernel density analysis of the economic loss.

Figure 8a shows the kernel density distribution curve of the health impact in 30 provinces in 2005, 2008, 2011, 2014, and 2017. In general, the distribution of the total health impact shows an obvious ‘multi-peak’ distribution in each period, indicating that there are significant differences in the total health impact of PM_2.5_ exposure in each province. We specifically divided the ‘multi-peak’ into the ‘main peak’, ‘second peak’, and ‘third peak’, implying low, medium, and high levels of health impact, respectively. During 2005–2014, the height of the ‘main peak’ of the health impact distribution curve continued to rise and shifted slightly to the right, indicating that the provinces with a low level of health impact increased, along with their health impact values. The ‘second peak’ gradually formed over time; the ‘third peak’ also showed a clear trend to the right, indicating an increase in the value of health impact in provinces with a high level of health impact. Overall, during 2005–2014, the value of health impact in most provinces showed an upward trend. In 2017, the height of the ‘main peak’ of the health impact distribution curve increased and shifted to the left, indicating that the provinces with a low level of health impact increased, and the value of health impact in these provinces decreased. The ‘second peak’ shifted to the right, but its peak value decreased; the ‘third peak’ shifted to the left, and the peak value increased, indicating that the provinces with a high level of health impact increased, but their health impact values decreased. Overall, the health impact in most provinces showed a downward trend during 2014–2017, with a notable polarisation phenomenon.

Figure 8b shows the kernel density distribution of economic losses in the 30 provinces. In general, the distribution of total economic loss shows a ‘double-peak’ distribution in each period. We divided the ‘double-peak’ into the ‘main peak’ and ‘second peak’, representing low and high levels of economic loss, respectively. During 2005–2014, the height of the ‘main peak’ of the economic loss distribution curve continued to decline and shifted slightly to the right, indicating that the provinces with a low level of economic loss decreased, and the value of economic loss in these provinces increased. The ‘second peak’ was constantly shifting to the right, indicating that the provinces with a high level of economic loss increased. Overall, the economic losses in most provinces increased from 2005–2014. Compared with 2014, the height of the ‘main peak’ of the economic loss distribution curve increased in 2017, indicating that the provinces with a low level of economic loss increased. The ‘second peak’ shifted to the left, indicating that the provinces with a high level of economic loss decreased. Overall, the economic losses of most provinces showed a downward trend in 2017.

### 3.4. Uncertainty Analysis

The difference in the PM_2.5_ baseline concentration will impact the estimation of health impact and economic loss due to PM_2.5_ exposure [57]. An amount of 10 µg/m^3^ was selected as the PM_2.5_ baseline concentration above. However, Quah and Boon [59] reported that there was no threshold value for haze particles on human health, and exposure to extremely low concentrations of PM_2.5_ would also damage human health. Therefore, we selected 0 µg/m^3^ as another baseline concentration and compared the corresponding results with previous studies. The comparison results of health impact and economic loss under different PM_2.5_ baseline concentrations during 2005–2017 are presented in Table 4 and Table 5, respectively. The reduction in the baseline concentration improves the estimation results of the health impact and economic loss. Compared with the baseline concentration of 10 µg/m^3^, the estimated health impact upon exposure to PM_2.5_ increased by approximately 28%, and the economic loss increased by approximately 30% when the reference concentration was 0 µg/m^3^. However, the proportion of economic loss in the GDP increased with a decrease in the baseline concentration. When the baseline concentration was 0 µg/m^3^, the proportion of economic loss in the GDP decreased from 2.72% in 2005 to 1.82% in 2017; when the baseline concentration was 10 µg/m^3^, the proportion of economic loss decreased from 2.07% in 2005 to 1.34% in 2017. The proportion of economic loss in the GDP increased by 0.51% annually when the baseline concentration dropped from 10 µg/m^3^ to 0 µg/m^3^.

## 4. Conclusions and Policy Implications

Accurately measuring the impact of PM_2.5_ on human health is key to more efficient pollution control and sustainable development. In this study, taking 30 provinces from 2005–2017 in China as examples, the health damage and economic loss of PM_2.5_ were measured, and its spatial and temporal evolution were analysed emphatically. The results show that an average of 15.03 million people were affected by PM_2.5_ each year, and the average annual economic loss was 86,886.94 million USD, accounting for 1.71% of the GDP. In terms of time trend, the health impact upon exposure to PM_2.5_ increased initially, then fluctuated to 2014 and peaked. The decline in health impact and economic loss after 2014 was due to the remarkable achievements of the Air Pollution Prevention and Control Action Plan implemented after 2013. At each health endpoint, the number of patients with acute bronchitis and chronic bronchitis accounted for 88%, and approximately 36% of the total economic loss. The all-cause mortality accounted for 1.5%; however, the economic loss accounted for 62%. Among them, lung cancer, cardiovascular disease, and respiratory disease are the main types of mortality due to PM_2.5_ exposure. Seasonally, the health impact and economic loss of PM_2.5_ exposure showed a U-shaped curve from spring to winter, which was in descending order: winter, spring, autumn, and summer; the health impact in winter was more than three times that in summer. Regarding the spatial distribution, East, North, Central, and South China are greatly affected by PM_2.5_, while Southwest, Northwest, and Northeast China are relatively weak; the spatial distribution showed a strong spatial correlation and significant polarisation phenomenon. Furthermore, after adjusting the baseline concentration from 10 to 0, the estimated health impact upon exposure to PM_2.5_ increased by approximately 28%, and the economic loss increased by approximately 30%.

The above conclusions highlight some critical policy implications. First, the decline in the health impact and economic loss after 2014 proves that more attention should be given to the positive impact of policy constraints on the prevention and control of air pollutants, and substantial support must be provided for top-level design to improve ecological performance and public welfare in the future. For example, increasing the use of natural gas, reducing vehicle quantity, and developing the tertiary industry can effectively mitigate the PM_2.5_ concentration. Second, given that the health impact and economic loss in winter is about 3.12 times and 3.29 times that in summer, and the monthly cases of health impact were the highest in January, effective measures must be taken to alleviate the problem of the rising PM_2.5_ concentration in winter especially in January, such as reducing coal consumption, and adopting more environmentally friendly and low-energy-consumption heating methods. Third, the health hazards of PM_2.5_ exposure had different characteristics in different provinces, also showing a high spatial correlation and significant polarisation phenomenon. Therefore, the implementation of regional joint prevention and control policy by taking provincial features into consideration are to be considered as important methods to effectively reduce the health damage caused by PM_2.5_. Finally, it is essential to improve the public knowledge and risk perception about PM_2.5_. On the one hand, this can prompt the public to pay more attention to pollution prevention for high-risk diseases due to PM_2.5_ exposure. On the other hand, the strengthening of public awareness is conducive to the implementation of environmental regulation policies for government [58,60], such as raising the prices of some types of energy and the environmental tax.

There are some directions for further research in the assessment of the health impact and economic loss by PM_2.5_ exposure. We only selected nine representative health endpoints strongly correlated with the health impact and did not consider other health endpoints such as obesity, diabetes, behaviour disorder, or the influence of genetic factors. In future research, more abundant health endpoints can be considered; not only that, other pollutants can also be included in the research system. For example, the impact of O_3_ pollution on public health is gaining more and more attention [61,62], and our following research will focus on investigating the synergistic impact of PM_2.5_ and O_3_ on human health to provide a reference for environmental governance and decision-making.

## Figures and Tables

**Table 1:** PM_2.5_ exposure-response coefficient of each health endpoint.

Health Endpoint	Exposure-Response Coefficient (β) (95% CI)	Reference
All-cause mortality	0.00090 (0.00000, 0.00180)	Cao et al. [42]; Huang et al. [43]; Yin et al. [38]
Respiratory mortality	0.00143 (0.00085, 0.00201)	Peng et al. [44]; Yin et al. [38]
Cardiovascular mortality	0.00053 (0.00015, 0.00090)	Peng et al. [44]; Yin et al. [38]
Lung cancer mortality	0.00340 (0.00000, 0.00710)	Cao et al. [42]; Huang et al. [43]; Yin et al. [38]
Respiratory hospital admission	0.00109 (0.00000, 0.00221)	Huang and Zhang [45]; Wang et al. [33]; Yang et al. [36]
Cardiovascular hospital admission	0.00068 (0.00043, 0.00093)	Huang and Zhang [45]; Wang et al. [33]; Yang et al. [36]; Yin et al. [38]
Chronic bronchitis	0.01009 (0.00366, 0.01559)	Huang and Zhang [45]; Wang et al. [46]; Wang et al. [33]
Acute bronchitis	0.00790 (0.00270, 0.01300)	Huang and Zhang [45]; Wang et al. [33]; Yin et al. [38]
Asthma attack	0.00210 (0.00145, 0.00274)	Huang and Zhang [45]; Yang et al. [36]; Yin et al. [38]

**Table 2:** Baseline incidence of each health endpoint.

Health Endpoint	Baseline Incidence (B)	Reference
All-cause mortality	0.006136	National Health and Family Planning Commission [47]
Respiratory mortality	0.000680	National Health and Family Planning Commission [47]
Cardiovascular mortality	0.002690	Yin et al. [38]
Lung cancer mortality	0.000497	Yin et al. [38]
Respiratory hospital admission	0.010200	National Health and Family Planning Commission [47]
Cardiovascular hospital admission	0.008550	Wang et al. [46]
Chronic bronchitis	0.006900	National Health and Family Planning Commission [47]
Acute bronchitis	0.038000	Yin et al. [38]
Asthma attack	0.009400	Yin et al. [38]

**Table 3:** Unit economic loss of each health endpoint in China in the base year (USD).

Health Endpoint	$\mathrm{Unit}\;\mathrm{Economic}\;\mathrm{Loss}\;(HC_{s,k})$	Method	Reference
All-cause mortality	132,000	Adjusted human capital (AHC)	Guo et al. [51]; Hammitt and Zhou, [52]; Yin et al. [38]
Respiratory mortality			
Cardiovascular mortality			
Lung cancer mortality			
Respiratory hospital admission	792.90	Cost of illness (COI)	Maji et al. [35]
Cardiovascular hospital admission	1600	Cost of illness (COI)	Yin et al. [38]
Chronic bronchitis	7000	Adjusted human capital (AHC)	Guo et al. [51]; Maji et al. [35]; Yin et al. [38]
Acute bronchitis	9	Willingness to pay (WTP)	Guo et al. [51]; Yin et al. [38]
Asthma attack	7	Willingness to pay (WTP)	Guo et al. [51]; Maji et al. [35]; Yin et al. [38]

**Table 4:** The comparison of health impact upon exposure to PM_2.5_ at different baseline concentrations.

Year	Baseline Concentration 0 µg/m^3^		Baseline Concentration 10 µg/m^3^	
	Health Impact (10^4^ Persons)	(95% Confidence Interval)	Health Impact (10^4^ Persons)	(95% Confidence Interval)
2005	1800.712	(695.872, 2685.142)	1392.344	(526.008, 2118.382)
2006	1978.924	(771.993, 2926.691)	1580.378	(602.849, 2383.763)
2007	2016.671	(787.824, 2978.886)	1617.336	(617.836, 2436.388)
2008	1962.916	(763.791, 2909.477)	1555.026	(591.624, 2351.009)
2009	1962.826	(763.109, 2911.493)	1550.647	(589.445, 2346.207)
2010	1966.307	(764.018, 2918.132)	1550.397	(589.000, 2347.077)
2011	1885.919	(729.167, 2810.957)	1461.029	(552.2416, 2221.853)
2012	1789.161	(687.764, 2680.348)	1353.639	(508.623, 2069.571)
2013	2041.683	(795.224, 3023.635)	1622.542	(617.935, 2450.876)
2014	2061.576	(803.365, 3051.796)	1640.878	(625.232, 2477.466)
2015	1956.514	(757.594, 2912.364)	1524.168	(576.990, 2314.685)
2016	1783.839	(684.017, 2678.245)	1334.372	(500.109, 2044.817)
2017	1801.503	(691.031, 2703.931)	1349.800	(506.072, 2067.790)

**Table 5:** The comparison of economic loss upon exposure to PM_2.5_ at different baseline concentrations.

Year	Baseline Concentration 0 µg/m^3^			Baseline Concentration 10 µg/m^3^		
	Economic Loss (million USD)	(95% Confidence Interval)	Proportion in GDP	Economic Loss (million USD)	(95% Confidence Interval)	Proportion in GDP
2005	57,522.02	(8660.47, 101,210.74)	2.72%	43,775.81	(6573.66, 77,554.43)	2.07%
2006	70,763.42	(10,663.94, 124,171.13)	2.87%	55,618.70	(8361.95, 98,241.52)	2.26%
2007	82,384.33	(12,416.80, 144,505.27)	2.78%	65,026.05	(9777.93, 114,807.88)	2.20%
2008	89,543.43	(13,490.81, 157,239.73)	2.47%	69,815.93	(10,493.16, 123,417.55)	1.92%
2009	96,165.01	(14,487.25, 168,908.35)	2.33%	74,771.21	(11,236.75, 132,212.59)	1.82%
2010	106,659.00	(16,067.26, 187,371.65)	2.27%	82,770.70	(12,438.028, 146,384.33)	1.76%
2011	112,843.20	(16,990.46, 198,519.83)	2.05%	86,041.52	(12,921.40, 152,408.87)	1.56%
2012	115,170.90	(17,330.35, 202,960.71)	1.85%	85,764.00	(12,869.78, 152,202.44)	1.38%
2013	142,425.00	(21,460.06, 250,030.15)	2.06%	111,397.80	(16,744.83, 196,863.19)	1.61%
2014	152,784.00	(23,021.99, 268,178.29)	2.02%	119,684.30	(17,991.49, 211,474.72)	1.58%
2015	151,470.60	(22,809.95, 266,359.28)	1.90%	116,137.40	(17,444.45, 205,620.39)	1.46%
2016	144,049.50	(21,669.92, 254,040.29)	1.80%	106,058.80	(15,909.77, 188,372.44)	1.33%
2017	152,775.30	(22,983.47, 269,400.71)	1.82%	112,668.00	(16,902.03, 200,088.18)	1.34%
